# Neonatal, infant, and under-5 mortality and morbidity burden in the Eastern Mediterranean region: findings from the Global Burden of Disease 2015 study

**DOI:** 10.1007/s00038-017-0998-x

**Published:** 2017-08-03

**Authors:** GBD 2015 Eastern Mediterranean Region Neonatal, Infant, and under-5 Mortality Collaborators, Ibrahim Khalil, Michael Collison, Charbel El Bcheraoui, Raghid Charara, Maziar Moradi-Lakeh, Ashkan Afshin, Kristopher J. Krohn, Farah Daoud, Adrienne Chew, Nicholas J Kassebaum, Danny Colombara, Leslie Cornaby, Rebecca Ehrenkranz, Kyle J. Foreman, Maya Fraser, Joseph Frostad, Laura Kemmer, Xie Rachel Kulikoff, Michael Kutz, Hmwe H. Kyu, Patrick Liu, Joseph Mikesell, Grant Nguyen, Puja C. Rao, Naris Silpakit, Amber Sligar, Alison Smith, Jeffrey D. Stanaway, Johan Ärnlöv, Kalkidan Hassen Abate, Aliasghar Ahmad Kiadaliri, Khurshid Alam, Deena Alasfoor, Raghib Ali, Reza Alizadeh-Navaei, Rajaa Al-Raddadi, Khalid A. Altirkawi, Nelson Alvis-Guzman, Nahla Anber, Hossein Ansari, Carl Abelardo T. Antonio, Palwasha Anwari, Al Artaman, Hamid Asayesh, Solomon Weldegebreal Asgedom, Peter Azzopardi, Umar Bacha, Aleksandra Barac, Suzanne L. Barker-Collo, Neeraj Bedi, Ettore Beghi, Derrick A. Bennett, Zulfiqar A. Bhutta, Donal Bisanzio, Carlos A. Castañeda-Orjuela, Ruben Estanislao Castro, Hadi Danawi, Kebede Deribe, Amare Deribew, Don C. Des Jarlais, Gabrielle A deVeber, Subhojit Dey, Samath D. Dharmaratne, Shirin Djalalinia, Huyen Phuc Do, Alireza Esteghamati, Maryam S Farvid, Seyed-Mohammad Fereshtehnejad, Florian Fischer, Tsegaye Tewelde Gebrehiwot, Giorgia Giussani, Philimon N. Gona, Nima Hafezi-Nejad, Randah Ribhi Hamadeh, Samer Hamidi, Damian G. Hoy, Guoqing Hu, Denny John, Jost B. Jonas, Seyed M. Karimi, Amir Kasaeian, Yousef Saleh Khader, Ejaz Ahmad Khan, Gulfaraz Khan, Daniel Kim, Yun Jin Kim, Yohannes Kinfu, Heidi J. Larson, Asma Abdul Latif, Janet L Leasher, Raimundas Lunevicius, Hassan Magdy Abd El Razek, Mohammed Magdy Abd El Razek, Azeem Majeed, Reza Malekzadeh, Ziad A. Memish, Walter Mendoza, Haftay Berhane Mezgebe, Ted R. Miller, Lorenzo Monasta, Quyen Le Nguyen, Carla Makhlouf Obermeyer, Alberto Ortiz, Christina Papachristou, Eun-Kee Park, Claudia C. Pereira, Max Petzold, Michael Robert Phillips, Farshad Pourmalek, Mostafa Qorbani, Anwar Rafay, Vafa Rahimi-Movaghar, Rajesh Kumar Rai, Saleem M Rana, David Laith Rawaf, Salman Rawaf, Andre M.N. Renzaho, Satar Rezaei, Mohammad Sadegh Rezai, Luca Ronfani, Gholamreza Roshandel, George Mugambage Ruhago, Mahdi Safdarian, Saeid Safiri, Mohammad Ali Sahraian, Payman Salamati, Abdallah M. Samy, Juan Ramon Sanabria, Benn Sartorius, David C. Schwebel, Soraya Seedat, Sadaf G. Sepanlou, Tesfaye Setegn, Amira Shaheen, Masood Ali Shaikh, Morteza Shamsizadeh, Rahman Shiri, Vegard Skirbekk, Badr H. A. Sobaih, Chandrashekhar T. Sreeramareddy, Vasiliki Stathopoulou, Rizwan Suliankatchi Abdulkader, Arash Tehrani-Banihashemi, Mohamad-Hani Temsah, JS Thakur, Alan J. Thomson, Bach Xuan Tran, Thomas Truelsen, Kingsley Nnanna Ukwaja, Olalekan A. Uthman, Tommi Vasankari, Vasiliy Victorovich Vlassov, Elisabete Weiderpass, Robert G. Weintraub, Andrea Werdecker, Mohsen Yaghoubi, Mehdi Yaseri, Naohiro Yonemoto, Mustafa Z. Younis, Chuanhua Yu, Aisha O. Jumaan, Theo Vos, Simon I. Hay, Mohsen Naghavi, Haidong Wang, Christopher J. L. Murray, Ali H. Mokdad

**Affiliations:** Institute for Health Metrics and Evaluation, Seattle, WA USA

**Keywords:** Child mortality, Burden of disease, Infant mortality, Neonatal mortality, Eastern Mediterranean Region

## Abstract

**Objectives:**

Although substantial reductions in under-5 mortality have been observed during the past 35 years, progress in the Eastern Mediterranean Region (EMR) has been uneven. This paper provides an overview of child mortality and morbidity in the EMR based on the Global Burden of Disease (GBD) study.

**Methods:**

We used GBD 2015 study results to explore under-5 mortality and morbidity in EMR countries.

**Results:**

In 2015, 755,844 (95% uncertainty interval (UI) 712,064–801,565) children under 5 died in the EMR. In the early neonatal category, deaths in the EMR decreased by 22.4%, compared to 42.4% globally. The rate of years of life lost per 100,000 population under 5 decreased 54.38% from 177,537 (173,812–181,463) in 1990 to 80,985 (76,308–85,876) in 2015; the rate of years lived with disability decreased by 0.57% in the EMR compared to 9.97% globally.

**Conclusions:**

Our findings call for accelerated action to decrease child morbidity and mortality in the EMR. Governments and organizations should coordinate efforts to address this burden. Political commitment is needed to ensure that child health receives the resources needed to end preventable deaths.

**Electronic supplementary material:**

The online version of this article (doi:10.1007/s00038-017-0998-x) contains supplementary material, which is available to authorized users.

## Introduction

Creating evidence-based estimates and understanding the causes of child mortality are essential for tracking progress toward child survival goals and for planning health strategies, policies, and interventions on child health. Substantial reductions have been observed in under-5 mortality worldwide during the past 35 years, with every region in the world recording sizeable improvements in child survival (Rajaratnam et al. 2010; Lozano et al. 2011; Wang et al. 2014; Liu et al. 2015; You et al. 2015).

The Global Burden of Disease (GBD) study provides an assessment of global child morbidity and mortality, documenting child health achievements during the Millennium Development Goal era and providing estimates of child mortality by age (neonatal, post-neonatal, 1–4 years, and under-5), sex, and cause over time (GBD 2015 Mortality and Causes of Death Collaborators 2016). In this manuscript, we used data from the GBD study to report child morbidity and mortality by age (neonatal, post-neonatal, 1–4 years, and under-5), sex, and cause over time in the EMR from 1990 to 2015.

This study provides the most comprehensive assessment so far of levels and trends of child morbidity and mortality in the EMR. Through a series of decomposition analyses, we identify which groups of causes contribute most to reductions in under-5 mortality across regions and the development spectrum. Comparisons of recorded levels and cause composition for child mortality by country offer an in-depth, nuanced picture of where countries might need to refocus policies and resource allocation to accelerate improvements in child survival in the future.

Millennium Development Goal 4 (MDG 4), “Reduce child mortality,” called for the reduction of the under-5 mortality rate by two-thirds between 1990 and 2015 (United Nations 2000). The new Sustainable Development Goals (SDGs) call for an end to preventable deaths of newborns and children by 2030, with all countries aiming to reduce neonatal mortality to at least as low as 12 per 1,000 live births and under-5 mortality to at least as low as 25 per 1000 live births (United Nations Sustainable Development Goals 2017). Globally, the number of under-5 deaths has declined by 52% (from 12.7 to 5.8 million from 1990 to 2015) (GBD 2015 Child Mortality Collaborators 2016), while progress across the EMR for child survival remains uneven. Nine countries (Bahrain, Egypt, Iran, Lebanon, Morocco, Oman, Saudi Arabia, Tunisia, and United Arab Emirates) met MDG 4 for annual reduction in child mortality of at least 4.4% between 1990 and 2015 in the EMR (GBD 2015 Child Mortality Collaborators 2016).

Neonatal deaths are the one of the largest causes of child mortality in the region, and are clearly linked to low levels of maternal health among the poorest segments of the population (Liu et al. 2012). The World Health Organization (WHO) and UNICEF reported that less than 50% of deliveries were attended by skilled health personnel in four countries—Afghanistan, Pakistan, Somalia, and Yemen—in the year 2010. Across the region, only 31% of married women use modern contraceptives, and 35% of newborns are delivered without a skilled birth attendant present (UNICEF and WHO 2012). Beyond the neonatal period, four disorders—diarrhea, pneumonia, malaria, and measles—are the major causes of post-neonatal death (Walker et al. 2013).

The Eastern Mediterranean Region (EMR) is home to more than 500 million people, representing a diverse group of 22 countries, including Arab states in North Africa, Gulf nations, and countries in West Asia; 12.2% of the population are children under 5 years of age, and 20% are women of childbearing age (WHO EMRO 2013).

EMR countries have diverse historical backgrounds, political and social contexts, and fiscal and cultural influences that impact maternal and child health. The region also has wide variation in per capita gross national product (GNP), ranging from a high of $134,420 in Qatar to a low of $2000 in Afghanistan (The World Bank GNI per capita 2017a).

While the Gulf States are some of the richest countries globally, poverty rates remain high in many other countries of the EMR. The proportion of the population living below the national poverty line, according to World Bank data, is more than 20% in seven EMR countries: Afghanistan (36%), Egypt (22%), Iraq (23%), Pakistan (22%), Palestine (22%), Sudan (47%), and Yemen (35%). In five of these countries, approximately one-third of the population is also food-insecure: Afghanistan (34%), Iraq (30%), Pakistan (30%), Sudan (33%), and Yemen (36%) (The World Bank Databank 2017b). Such wide variation has a major influence on overall health spending and results in substantial health inequities both within and across countries.

## Methods

The methods used to generate estimates of under-5 mortality and age-specific death rates (neonatal, post-neonatal, ages 1–4 years, and under-5), contribute to broader GBD 2015 analyses and results on all-cause mortality and cause of death. Substantial detail on data inputs, processing, and estimation methods can be found elsewhere (GBD 2015 Mortality and Causes of Death Collaborators 2016). Here, we provide a brief summary of our under-5 mortality estimation approach and accompanying analyses, including an assessment of mortality trends by Socio-demographic Index (SDI), and changes in under-5 mortality attributable to leading causes of death.

Our GBD 2015 analyses follow the recently proposed Guidelines for Accurate and Transparent Health Estimates Reporting (GATHER) (Stevens et al. 2016), which include the documentation of data sources and inputs, processing and estimation steps, and overarching methods used throughout the GBD study.

### Data

Data sources and types used for estimating child mortality are described extensively elsewhere (GBD 2015 Mortality and Causes of Death Collaborators 2016), but in sum, vital registration (VR) systems, censuses, and household surveys with complete or summary birth histories served as primary inputs for our analyses. Other sources, including sample registration systems and disease surveillance systems, also contributed as input data. In total we applied formal demographic techniques to 8169 input data sources of under-5 mortality from 1950 to 2015. Overall data availability and availability by source data type varied by country.

### All-cause under-5 mortality and age-specific mortality

We estimated all-cause under-5 mortality and death rates by age group: neonatal (0–28 days), post-neonatal (29–364 days), and ages 1–4 years. Details on data bias adjustments for under-5 mortality, using spatiotemporal Gaussian process regression to generate a complete time series of under-5 mortality for EMR countries and the age–sex model to produce estimates of mortality for neonatal, post-neonatal, and ages 1–4 years, have been extensively discussed previously (Wang et al. 2014).

To estimate mortality by age group and sex within the under-5 categorization, we used a two-stage modeling process that has been described in detail elsewhere (GBD 2015 Mortality and Causes of Death Collaborators 2016). For this analysis, we report on early neonatal and late neonatal mortality results in aggregate as neonatal mortality.

### Under-5 causes of death

The methods developed and used in GBD 2015, including the systematic approach to collating causes of death from different countries; mapping across different revisions; redistributing deaths assigned to so-called garbage codes; and the overall and disease-specific cause of death modeling approaches, are described in other publications (Foreman et al. 2012; GBD 2015 Mortality and Causes of Death Collaborators 2016).

For GBD 2015, we assessed 249 causes of death across age groups. Because of cause-specific age restrictions (e.g., no child deaths due to Alzheimer’s disease and other dementias), not all causes of death were applicable for children younger than 5 years (GBD 2015 Mortality and Causes of Death Collaborators 2016).

### YLLs, YLDs, and DALYs

We calculated years of life lost (YLLs) by multiplying deaths by the remaining life expectancy at the age of death from a standard life table chosen as the norm for estimating premature mortality in GBD. We consider the standard life expectancy as a composite of the best case mortality scenario for every year, age, and sex. The metric therefore highlights premature deaths by applying a larger weight to deaths that occur at younger ages. Years lived with disability (YLDs) were calculated by multiplying the number of prevalent cases of a certain health outcome by the disability weight assigned to this health outcome. A disability weight reflects the magnitude of the health loss associated with an outcome and has a value that is anchored between 0, equivalent to full health, and 1, equivalent to death. Disability-adjusted life years (DALYs) were calculated by adding YLLs and YLDs. Detailed methods on YLLs, YLDs, and DALYs are published elsewhere (GBD 2015 Disease and Injury Incidence and Prevalence Collaborators 2016; Kassebaum et al. 2016).

### Socio-demographic Index

We studied patterns in child mortality as they related to measures of socioeconomic status and development. Drawing on methods used to construct the Human Development Index (HDI) (UNDP 2016), we created a composite indicator, the Socio-demographic Index (SDI), based on equally weighted estimates of lagged distributed income (LDI) per person, average years of education among individuals older than 15 years, and total fertility rate. SDI was constructed as the geometric mean of these three values. To capture the average relationships for each age–sex group, we applied a simple least squares spline regression of mortality rate on SDI. SDI values were scaled to a range of 0–1, with 0 equaling measures of the lowest educational attainment, lowest income, and highest fertility rate between 1980 and 2015, and 1 equaling measures of the highest educational attainment, highest income, and lowest fertility rate during this time. Additional information can be found elsewhere (GBD 2015 Mortality and Causes of Death Collaborators 2016).

### Decomposing change in under-5 mortality rate by causes of death

Based on the age-specific, sex-specific, and cause-specific mortality results from GBD 2015 (GBD 2015 Mortality and Causes of Death Collaborators 2016), we attributed changes in under-5 mortality rate between 1990 and 2015 to changes in leading causes of death in children younger than 5 years in the EMR during the same period. To do this, we applied the decomposition method developed by Beltran-Sanchez and colleagues (Beltran-Sanchez et al. 2008), which has also been used for other GBD analyses (GBD 2015 Mortality and Causes of Death Collaborators 2016).

### Uncertainty analysis

We propagated known measures of uncertainty through key steps of the mortality estimation processes, including uncertainty associated with varying sample sizes of data, source-specific adjustments to data used for all-cause mortality, model specifications for spatiotemporal Gaussian process regression (ST-GPR) and cause-specific model specifications, and estimation procedures. Uncertainty estimates were derived from 1000 draws for under-5 mortality, age-specific mortality, and cause-specific mortality by sex, year, and geography from the posterior distribution of each step of the estimation process. These draws allowed us to quantify, and then propagate, uncertainty for all mortality metrics. Percent changes and annualized rates of change were calculated between mean estimates, while the uncertainty intervals associated with the percent changes were derived from the 1000 draws.

## Results

### Mortality

All-cause mortality rates for under-5 age groups in the EMR decreased from 1990 to 2015, closely following global patterns of decline of around 54% (Institute for Health Metrics and Evaluation 2017). In 2015, there were 755,843 under-5 deaths in the EMR, which constitute about 18.8% of total deaths in the region for all ages. The largest difference in under-5 deaths was in the early neonatal category, where deaths in the EMR decreased by 22.4%, in comparison to 42.4% globally (Institute for Health Metrics and Evaluation 2017). Total deaths for all under-5 age groups decreased in the EMR at a slower rate than globally (e-Table 1).

In 2015, neonatal mortality was the largest contributing group to under-5 mortality in most EMR countries (Table 1). The exceptions to this were Afghanistan, Djibouti, and Syria, with roughly equal mortality rates for neonatal, post-neonatal, and child (1–4 years) age groups, and Somalia with a child mortality rate of 44.6 (95% UI: 32.4–58.8) deaths per 1000 live births compared to a neonatal mortality rate of 31.3 (27.2–35.9) (Table 1). Somalia also had the highest under-5 mortality rate of 112.2 (97.5–130.4) deaths per 1000 live births. The United Arab Emirates had the lowest under-5 mortality rate, 5.5 (3.2–9.1) deaths per 1000 live births. Under-5 mortality rate declined annually from 1990 to 2015 in all countries, ranging from Somalia with the smallest rate of change 2.1 (1.4–2.7) to Iran with the largest 6.5 (5.2–7.9).

**Table 1:** Mortality rates, deaths and annual rate of decline in mortality by country in 2015

Country	Deaths per 1000 livebirths				Total under-5 deaths (thousands)	Annualized rate of decline for under-5 mortality		
	Neonatal (0–27 days)	Post-neonatal (28 days–12 months)	Child (12–59 months)	Under-5		1990–2000	2000–2015	1990–2015
Global	12.2 (11.0–13.6)	12.2 (10.9–13.6)	11.2 (9.8–12.8)	41.4 (37.9–45.5)	5820.9 (5673.3–5965.1)	2.0 (1.7–2.4)	3.6 (3.0–4.2)	3.0 (2.6–3.3)
EMR	22.5 (21.1–24.0)	12.4 (11.4–13.4)	9.3 (8.1–10.5)	44.2 (41.6–46.9)	755.8 (712.1–801.6)	–	–	–
Afghanistan	28.6 (24.7–33.0)	30.5 (24.0–38.2)	25.9 (17.3–36.3)	82.6 (69.5–98.0)	89.3 (75.5–105.6)	5.1 (4.1–6.2)	4.3 (3.2–5.6)	2.7 (1.9–3.5)
Bahrain	3.8 (3.2–4.5)	2.0 (1.6–2.3)	0.9 (0.6–1.2)	6.6 (5.6–7.8)	0.1 (0.1–0.2)	1.0 (0.2–1.8)	3.0 (2.0–3.9)	4.7 (4.0–5.3)
Djibouti	23.4 (20.3–27.0)	23.2 (18.1–29.1)	20.4 (14.0–28.7)	65.5 (55.4–78.7)	1.4 (1.2–1.7)	6.4 (5.5–7.4)	4.7 (2.8–6.6)	2.2 (1.4–2.9)
Egypt	12.1 (8.9–15.4)	6.0 (4.3–8.6)	3.6 (2.6–5.1)	21.5 (16.3–28.3)	53.3 (40.1–70.1)	6.5 (4.2–8.9)	6.5 (4.1–8.9)	5.4 (4.2–6.5)
Iran	8.1 (5.6–11.4)	4.0 (2.9–5.3)	2.7 (1.7–3.9)	14.7 (10.8–19.5)	19.9 (14.7–26.5)	2.4 (1.7–3.0)	2.9 (1.7–4.1)	6.5 (5.2–7.9)
Iraq	15.5 (13.4–18.0)	7.1 (5.6–9.1)	5.0 (3.3–7.2)	27.3 (23.2–32.6)	33.5 (28.9–39.2)	2.9 (2.4–3.5)	3.3 (2.3–4.2)	2.7 (2.0–3.4)
Jordan	7.7 (6.4–9.2)	3.5 (2.9–4.2)	3.6 (2.6–4.6)	14.8 (12.9–17.2)	2.9 (2.6–3.4)	2.5 (1.2–3.8)	2.9 (1.3–4.5)	3.1 (2.6–3.7)
Kuwait	4.4 (3.4–5.4)	2.4 (1.8–3.2)	1.4 (1.0–2.0)	8.2 (6.5–10.2)	0.6 (0.5–0.8)	4.8 (2.3–7.5)	6.6 (4.0–9.0)	2.7 (1.7–3.8)
Lebanon	4.4 (3.2–6.0)	2.2 (1.6–3.2)	1.5 (1.0–2.3)	8.1 (5.9–11.3)	0.7 (0.5–0.9)	3.9 (2.5–5.4)	3.2 (1.6–4.7)	5.9 (4.5–7.3)
Libya	8.1 (5.7–11.1)	4.7 (3.5–6.3)	4.8 (3.23–6.9)	17.5 (13.2–22.6)	2.3 (1.8–3.0)	4.7 (3.8–5.5)	4.5 (3.2–5.8)	3.5 (2.3–4.6)
Morocco	12.7 (10.0–15.5)	5.8 (4.3–7.8)	4.1 (3.0–5.6)	22.4 (18.0–27.9)	15.7 (12.6–19.5)	10.2 (8.6–11.0)	3.3 (2.0–4.6)	4.6 (3.7–5.5)
Oman	4.7 (4.1–5.3)	2.6 (2.1–3.2)	2.1 (1.6–2.8)	9.4 (8.1–10.8)	0.8 (0.7–0.9)	2.0 (1.6–2.4)	2.4 (1.8–3.1)	6.0 (5.2–6.9)
Pakistan	37.9 (34.8–41.3)	15.9 (13.6–18.5)	10.4 (7.3–13.8)	63.0 (57.4–69.4)	341.7 (311.3–376.0)	4.2 (2.7–5.8)	2.7 (1.0–4.5)	2.3 (1.9–2.7)
Palestine	9.9 (7.3–12.7)	4.6 (3.7–6.0)	2.9 (2.0–4.0)	17.3 (13.5–21.8)	2.6 (2.0–3.3)	3.0 (1.2–7.0)	4.2 (1.1–7.1)	3.3 (2.3–4.4)
Qatar	4.7 (3.2–6.6)	2.4 (1.6–3.5)	1.6 (1.0–2.4)	8.6 (6.0–12.1)	0.2 (0.2–0.3)	6.7 (5.0–8.3)	4.9 (3.1–6.6)	3.7 (2.0–5.7)
Saudi Arabia	6.1 (4.4–9.2)	3.2 (2.2–4.4)	2.2 (1.4–3.2)	11.5 (8.3–16.3)	7.1 (6.3–8.1)	1.7 (1.0–2.3)	2.3 (1.4–3.2)	5.6 (4.2–6.9)
Somalia	31.3 (27.2–35.9)	40.8 (32.8–49.6)	44.6 (32.4–58.8)	112.2 (97.5–130.4)	51.7 (44.7–60.3)	3.2 (2.0–4.3)	3.7 (1.8–5.5)	2.1 (1.4–2.7)
Sudan	24.1 (19.9–29.4)	17.2 (12.4–23.4)	15.8 (10.4–23.8)	56.0 (43.3–73.7)	73.2 (56.7–96.3)	7.0 (6.1–7.9)	0.4 (2.4–1.8)	3.5 (2.4–4.6)
Syria	7.1 (6.0–8.5)	5.8 (4.3–7.5)	9.7 (4.9–15.5)	22.4 (16.2–29.3)	10.1 (7.3–12.9)	6.0 (5.2–7.0)	4.5 (3.3–5.7)	2.6 (1.4–3.9)
Tunisia	7.4 (5.9–9.2)	3.5 (2.9–4.2)	3.0 (2.1–3.9)	13.8 (11.5–16.5)	2.8 (2.3–3.3)	6.8 (0.1–13.0)	5.4 (0.5–9.9)	5.1 (4.3–5.8)
United Arab Emirates	2.9 (1.5–5.0)	1.5 (1.0–2.5)	1.1 (0.6–1.7)	5.5 (3.2–9.1)	0.5 (0.3–0.9)	5.1 (4.1–6.2)	4.3 (3.2–5.6)	6.0 (2.9–8.7)
Yemen	20.9 (19.1–23.2)	18.0 (14.9–21.9)	15.7 (10.3–22.6)	53.6 (45.9–63.2)	45.5 (40.2–51.3)	1.0 (0.2–1.8)	3.0 (2.0–3.9)	2.7 (1.9–3.5)

95% uncertainty intervals are provided in parentheses. Annualized rate of decline not available for the Eastern Mediterranean Region in aggregate. (Global Burden of Disease 2015 Study, Global, Eastern Mediterranean Countries, 1990–2015)

**Fig. 1:**
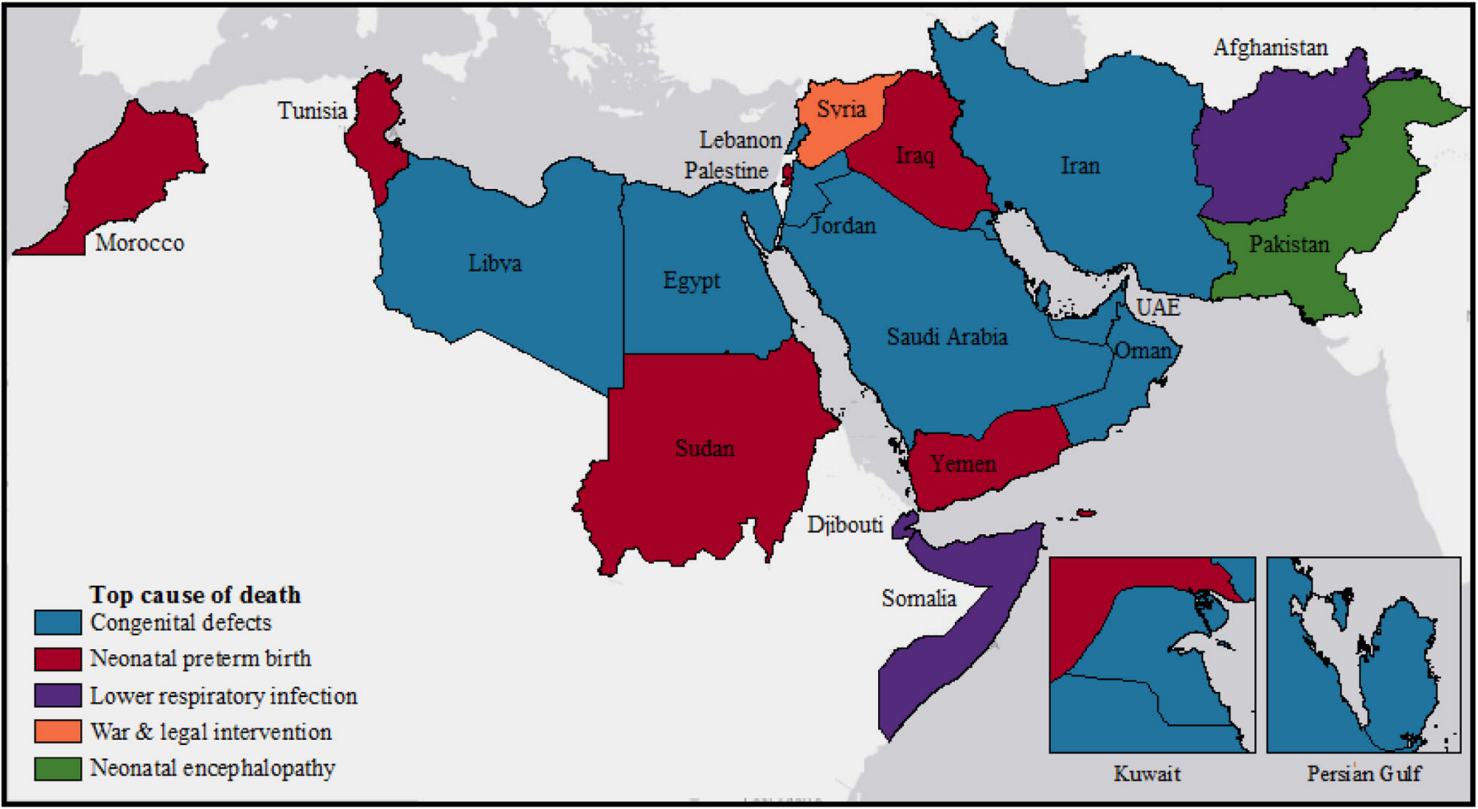
Top cause of under-5 deaths in the Eastern Mediterranean Region by country, 2015 (Global Burden of Disease 2015 study, Eastern Mediterranean Countries, 2015)

**Fig. 2:**
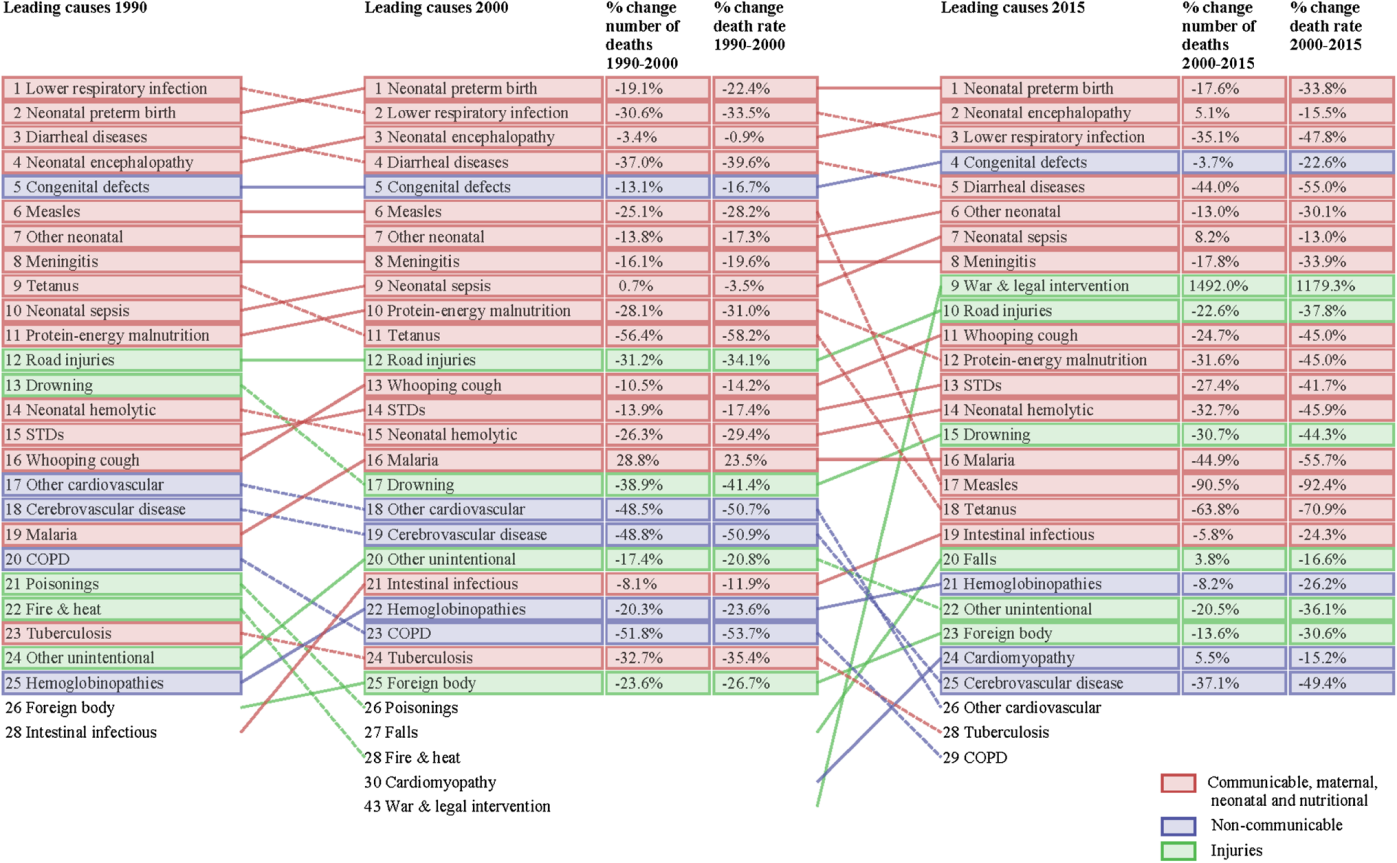
Changes in number of deaths and mortality rates in top 25 causes of under-5 mortality in the Eastern Mediterranean Region, 1990–2000 and 2000–2015. Data available at https://vizhub.healthdata.org/gbd-compare. (Global Burden of Disease 2015 study, Eastern Mediterranean Region, 1990–2015)

Figure 1 shows the top cause of under-5 mortality for individual countries in 2015. The top five causes of under-5 mortality—preterm birth complications, neonatal encephalopathy, lower respiratory infections, congenital defects, and diarrheal disease—were the same in the EMR and globally, with congenital defects and diarrheal diseases ranked fourth and fifth in the EMR, but fifth and fourth globally (Institute for Health Metrics and Evaluation 2017). War ranked ninth in the EMR and 25th globally (Fig. 2). From 1990 to 2015, the top five causes of under-5 mortality in the EMR remained the same. War moved from 43rd to ninth between 2000 and 2015, and measles dropped from sixth to 17th.

**Fig. 3:**
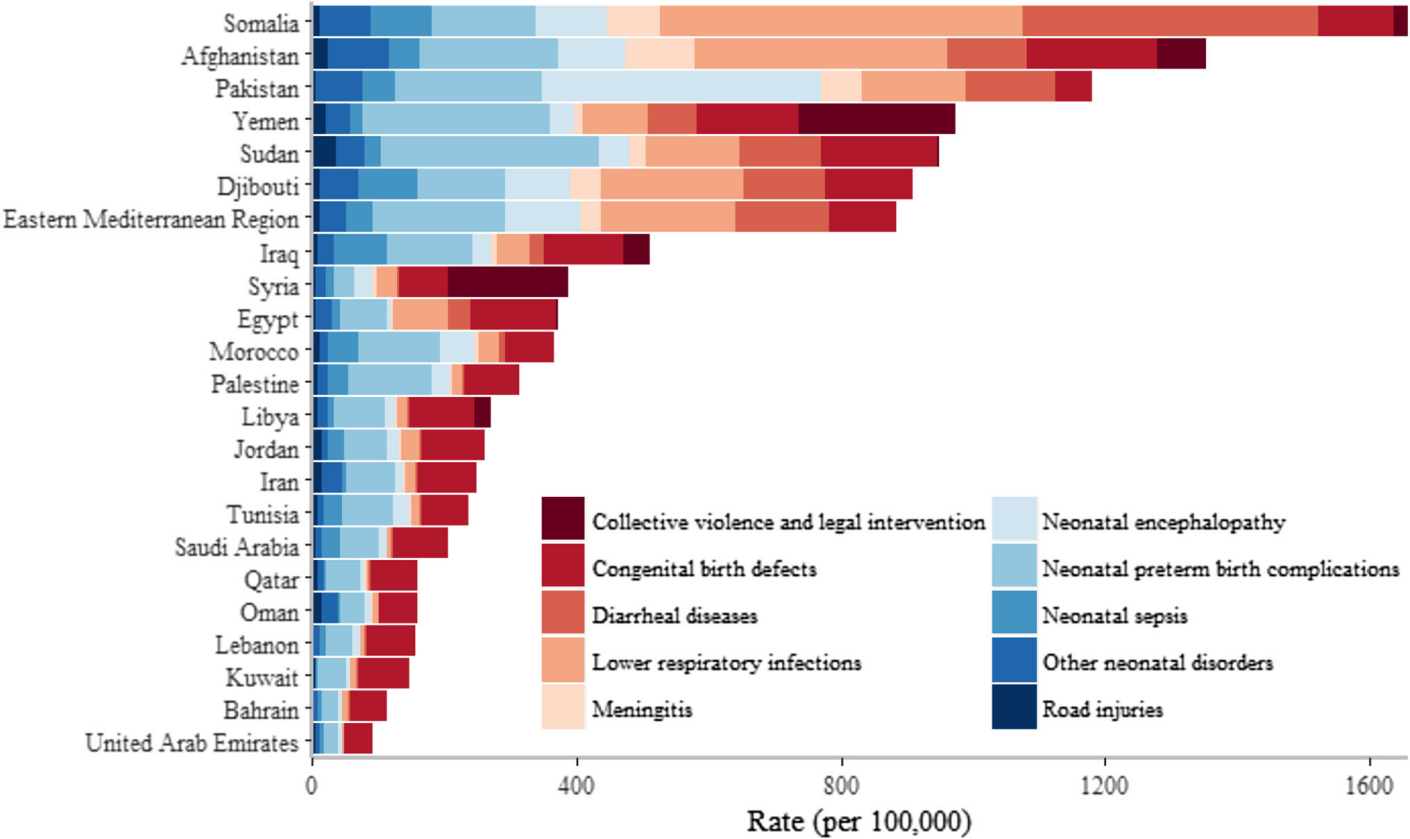
Mortality rates for top 10 causes of under-5 mortality in the Eastern Mediterranean Region by country, 2015 (Global Burden of Disease 2015 study, Eastern Mediterranean Countries, 2015)

In Afghanistan, mortality rates from nine top-10 causes were greater than the EMR average, with mortality from neonatal encephalopathy as the only exception (Table 2). Likewise, all countries except Pakistan fell beneath the average regional rate for neonatal encephalopathy, with a rate of 423.6 (318.5–528.3) per 100,000 population under 5 compared to the regional rate of 154.4 (121.7–187.9). Bahrain, Kuwait, Lebanon, Palestine, Qatar, Saudi Arabia, Tunisia, and United Arab Emirates were below the average regional rates in all top-10 causes. Somalia, Afghanistan, and Pakistan had the highest mortality rates for the top 10 sub-causes of under-5 morality in 2015, while United Arab Emirates, Bahrain, and Kuwait had the lowest (Fig. 3).

**Table 2:** Mortality rates for 10 major causes of under-5 mortality by country in 2015

Country	Neonatal preterm birth	Neonatal encephalopathy	Lower respiratory infection	Congenital defects	Diarrheal diseases	Other neonatal	Neonatal sepsis	Meningitis	War and legal intervention	Road injuries
Global	120.0 (109.6–133.8)	110.3 (99.4–123.5)	104.82 (97.0–113.6)	74.0 (66.2–82.6)	74.3 (66.6–83.0)	32.8 (25.0–41.2)	52.37 (37.11–68.37)	25.8 (20.4–34.1)	3.5 (2.2–4.8)	7.4 (6.4–8.5)
Eastern Mediterranean Region	163.6 (136.0–195.6)	154.4 (121.7–187.9)	122.7 (106.9–140.8)	102.7 (86.4–128.2)	81.8 (66.8–99.0)	43.5 (27.2–64.5)	36.4 (19.1–57.9)	31.9 (22.4–46.9)	26.1 (16.4–36.4)	11.8 (8.9–15.8)
Afghanistan	209.6 (137.9–301.0)	103.4 (59.1–161.9)	380.0 (254.4–518.7)	198.5 (100.2–433.4)	122.0 (71.7–188.5)	93.1 (31.2–180.2)	45.9 (17.6–91.3)	104.6 (53.4–191.1)	72.6 (25.6–119.9)	23.4 (9.4–49.1)
Bahrain	25.5 (19.7–32.3)	6.2 (4.4–8.3)	8.6 (6.0–11.2)	54.2 (41.2–71.2)	3.0 (1.9–4.1)	6.2 (4.3–8.5)	5.4 (3.0–7.9)	0.8 (0.5–1.2)	–	2.5 (1.5–3.8)
Djibouti	130.2 (79.1–189.4)	98.8 (50.1–162.1)	215.8 (134.6–301.9)	134.0 (85.9–187.4)	122.9 (59.1–193.1)	60.6 (23.0–117.5)	90.0 (44.8–165.5)	47.0 (20.7–89.8)	–	9.8 (3.0–23.5)
Egypt	72.4 (51.2–96.8)	6.8 (4.0–11.4)	83.0 (59.7–113.5)	130.3 (91.0–177.3)	34.2 (23.0–52.1)	22.0 (13.8–32.4)	12.5 (5.4–20.4)	2.4 (1.7–3.6)	1.7 (0.6–2.8)	6.3 (4.2–9.2)
Iran	73.1 (45.0–109.6)	13.5 (6.7–24.1)	17.1 (11.1–25.6)	87.1 (56.0–125.2)	3.5 (1.9–6.1)	29.9 (15.1–51.7)	6.1 (2.3–12.3)	2.4 (1.2–4.2)	–	14.8 (7.9–24.1)
Iraq	127.7 (85.9–168.5)	27.9 (14.8–46.5)	47.1 (32.7–63.7)	121.5 (78.8–209.8)	21.7 (12.7–33.4)	23.4 (9.6–46.6)	81.6 (32.2–137.8)	11.4 (5.2–22.0)	39.2 (13.8–64.7)	9.0 (3.7–20.0)
Jordan	65.9 (48.6–85.8)	19.8 (12.2–29.8)	28.2 (21.8–36.4)	95.3 (78.4–115.6)	2.5 (1.6–3.9)	9.0 (4.6–16.5)	23.9 (12.9–41.4)	2.9 (1.6–5.0)	–	13.8 (7.8–20.8)
Kuwait	42.4 (32.0–54.4)	4.7 (3.4–6.4)	10.1 (7.6–13.7)	76.8 (59.1–99.2)	1.1 (0.8–1.6)	2.6 (1.8–3.6)	2.4 (1.4–4.6)	1.2 (0.9–1.6)	0.3 (0.1–.6)	4.6 (3.2–6.4)
Lebanon	40.3 (25.0–59.4)	11.4 (5.6–19.8)	6.8 (3.9–11.1)	74.4 (45.8–108.9)	1.8 (0.8–3.5)	8.4 (3.1–17.6)	8.9 (3.7–17.2)	1.4 (0.5–3.4)	1.1 (0.4–1.8)	2.4 (0.9–5.8)
Libya	77.7 (50.7–113.7)	15.0 (7.6–25.8)	15.1 (10.0–22.0)	95.9 (70.7–128.3)	4.9 (2.5–8.2)	12.6 (4.9–25.6)	10.7 (4.4–21.5)	2.8 (1.2–4.9)	25.5 (9.0–42.0)	9.5 (4.6–17.1)
Morocco	124.8 (83.2–169.8)	50.3 (31.2–75.7)	29.0 (19.8–41.5)	75.9 (45.5–141.8)	8.6 (5.2–13.6)	10.7 (4.8–20.4)	46.7 (26.0–75.9)	7.9 (4.5–13.5)	–	12.5 (6.8–20.8)
Oman	36.0 (25.4–47.4)	10.1 (5.9–15.6)	9.6 (6.9–13.1)	57.1 (44.0–74.4)	1.0 (0.6–1.6)	26.5 (15.5–38.8)	2.4 (0.5–6.1)	1.4 (0.8–2.8)	–	14.2 (8.4–21.7)
Pakistan	221.7 (140.1–309.8)	423.6 (318.5–528.3)	157.7 (118.9–200.8)	56.3 (39.5–72.9)	135.3 (92.6–185.8)	68.4 (27.9–126.4)	50.6 (20.8–107.9)	60.4 (35.5–102.5)	1.4 (0.7–2.1)	6.5 (2.4–14.9)
Palestine	126.2 (91.3–165.7)	28.5 (18.1–43.4)	14.2 (9.6–20.4)	84.8 (56.8–140.0)	2.3 (1.5–3.5)	14.3 (7.2–25.9)	31.6 (5.1–56.7)	2.4 (1.3–3.8)	–	9.4 (4.8–15.6)
Qatar	53.3 (35.1–79.9)	8.5 (4.5–14.8)	3.9 (2.3–6.3)	73.5 (47.7–107.0)	0.8 (0.4–1.4)	10.0 (4.9–17.4)	1.2 (0.5–2.4)	1.6 (0.8–2.9)	–	7.9 (3.9–13.5)
Saudi Arabia	58.1 (40.4–71.7)	12.8 (8.8–16.5)	4.3 (3.5–5.3)	84.9 (70.1–109.4)	3.4 (2.6–4.3)	8.6 (5.0–12.2)	27.6 (20.7–40.7)	0.8 (0.5–1.2)	0.1 (0.0–0.4)	5.9 (4.4–8.1)
Somalia	158.0 (95.8–235.9)	106.5 (54.0–176.6)	546.8 (404.5–716.4)	114.2 (81.9–155.5)	449.6 (296.7–630.6)	78.0 (26.1–165.6)	91.2 (40.6–168.5)	82.1 (43.2–144.7)	21.2 (.0–54.4)	11.6 (4.7–26.3)
Sudan	330.1 (249.5–419.0)	45.9 (23.7–79.9)	142.2 (89.8–218.9)	178.0 (104.6–323.2)	124.8 (70.0–205.2)	42.2 (17.1–87.2)	24.6 (10.8–52.9)	24.8 (10.3–45.6)	2.0 (0.0–5.5)	35.8 (12.1–73.7)
Syria	30.9 (20.1–44.1)	27.5 (14.5–45.0)	32.5 (22.8–42.4)	73.6 (52.6–89.8)	2.2 (1.3–3.5)	15.4 (5.6–28.7)	12.9 (6.3–23.8)	6.2 (3.2–10.7)	180.0 (63.6–297.1)	5.0 (2.3–9.2)
Tunisia	76.9 (55.3–105.1)	25.9 (16.1–38.7)	11.3 (8.1–15.2)	70.4 (52.9–97.8)	3.8 (2.3–5.7)	11.9 (5.8–21.5)	25.5 (14.4–40.9)	4.0 (2.3–6.8)	0.7 (0.2–1.2)	6.7 (3.6–11.5)
United Arab Emirates	21.0 (10.5–38.6)	6.8 (2.6–14.2)	2.1 (1.1–3.9)	42.1 (22.1–72.5)	0.5 (0.2–1.0)	6.1 (2.2–13.7)	7.2 (2.8–14.9)	1.4 (0.6–3.0)	–	4.5 (1.9–8.9)
Yemen	281.4 (204.2–352.9)	38.4 (19.2–66.3)	100.2 (69.1–138.9)	152.0 (94.5–249.3)	74.3 (37.8–120.0)	38.4 (13.1–83.8)	18.2 (7.1–37.7)	12.1 (4.5–22.4)	239.5 (148.0–331.0)	20.4 (8.5–39.1)

All rates are per 100,000 population under-5. 95% uncertainty intervals are provided in parentheses. War and legal intervention was left empty where values were not estimated. (Global Burden of Disease 2015 Study, Global, Eastern Mediterranean Countries, 1990–2015)

### Observed mortality versus expected mortality based on SDI alone

Observed mortality rates in the EMR have been consistently lower than expected mortality rates based on SDI alone for the under-5 age group (e-Fig. 1). Kuwait had the highest observed-to-expected ratio at 1.61, followed by United Arab Emirates at 1.15 (e-Table 2). Kuwait and United Arab Emirates have the highest SDIs in the region, at 0.86 and 0.88, respectively. Djibouti, Pakistan, and Qatar also had observed-to-expected ratios greater than 1. Morocco and Palestine had the lowest ratios at 0.42 and 0.44, respectively, with SDIs at 0.5 and 0.57. Somalia, with the lowest SDI in the region, had a ratio of 0.58.

### YLLs

The decrease in YLL rate per 100,000 population under 5 from 1990 to 2015 was similar globally and for the EMR, with percent decreases of about 54% (Table 3). From 1990 to 2015, YLLs decreased in all countries (Table 3). The largest decrease was in Iran, where the YLL rate decreased 81% from 132,265 (116,751–150,030) to 25,276 (18,585–33,780) per 100,000 population under 5. The smallest decrease was in Kuwait, where the YLL rate decreased 42% from 25,451 (22,873–28,223) to 14,665 (11,594–18,408) per 100,000 population under 5. Similarly, Somalia’s YLL rate decreased 43% from 380,035 (359,276–402,133) to 217,737 (188,533–253,963) per 100,000 population under 5.

**Table 3:** YLLs, YLDs, and DALYs per 100,000 under-5 population and percent change by country, 1990–2015

Country	SDI (2015)	Under-5 YLL rate per 100,000			Under-5 YLD rate per 100,000			Under-5 daly rate per 100,000		
		1990	2015	% change	1990	2015	% change	1990	2015	% change
Global	0.64	162,811 (161,334–164,422)	74,441 (72,554–76,285)	−54	5512 (3868–7521)	4962 (3499.8–6746)	−10	168,324 (166,143–170,828)	79,403 (76,806–81,838)	−53
Eastern Mediterranean Region	0.55	177,537 (173,812–181,463)	80,985 (76,308–85,876)	−54	5388 (3866–7271)	5357 (3750–7290)	−1	182,925 (178,760–186,979)	86,342 (81,261–91,567)	−53
Afghanistan	0.29	326,350 (303,452–351,586)	152,191 (128,892–180,117)	−53	6598 (4553–9151)	5530 (3849–7565)	−16	332,948 (310,338–358,029)	157,721 (134,351–185,824)	−53
Bahrain	0.78	37,616 (35,223–40,144)	11,413 (9697–13,368)	−70	4006 (2826–5397)	3374 (2378–4606)	−16	41,622 (38,741–44,523)	14,787 (12,824–17,208)	−64
Djibouti	0.46	226,135 (210,850–241,280)	118,367 (99,999–142,052)	−48	6492 (4552–8834)	6743 (4588–9207)	4	232,627 (217,252–247,793)	125,110 (106,641–148,413)	−46
Egypt	0.62	151,318 (142,553–159,885)	39,029 (29,380–51,379)	−74	4960 (3516–6837)	4496 (3053–6390)	−9	156,278 (147,486–164,909)	43,525 (34,043–56,032)	−72
Iran	0.72	132,265 (116,751–150,030)	25,276 (18,585–33,780)	−81	5616 (4016–7677)	3864 (2709–5313)	−31	137,881 (122,316–155,406)	29,140 (22,262–37,880)	−79
Iraq	0.58	101,180 (96,461–105,998)	50,346 (43,459–59,054)	−50	4818 (3435–6478)	4596 (3167–6566)	−5	105,997 (100,973–110,991)	54,941 (47,877–63,758)	−48
Jordan	0.7	60,426 (58,011–62,916)	26,220 (22,899–30,491)	−57	3923 (2755–5337)	3466 (2427–4732)	−12	64,349 (61,841–67,152)	29,686 (26,108–33,991)	−54
Kuwait	0.86	25,451 (22,873–28,223)	14,665 (11,594–18,408)	−42	3924 (2724–5617)	3010 (2103–4102)	−23	29,374 (26,334–32,511)	17,675 (14,368–21,513)	−40
Lebanon	0.75	61,129 (52,841–70,642)	15,562 (11,368–21,614)	−75	6804 (4457–10,960)	3878 (2676–5307)	−43	67,933 (59,255–77,662)	19,440 (15,114–25,648)	−71
Libya	0.64	72,412 (65,190–79,811)	29,599 (22,636–38,322)	−59	3940 (2767–5607)	4229 (2828–6079)	7	76,352 (69,519–83,925)	33,828 (26,576–42,261)	−56
Morocco	0.5	126,475 (121,222–131,669)	39,544 (31,692–49,221)	−69	4975 (3456–6725)	4207 (2936–5730)	−15	131,450 (126,095–137,032)	43,751 (35,664–53,401)	−67
Oman	0.73	75,607 (63,659–90,351)	17,135 (14,831–19,666)	−77	5879 (4039–8106)	4868 (3326–6827)	−17	81,486 (69,237–96,665)	22,002 (19,103–24,919)	−73
Pakistan	0.47	217,582 (211,203–224,200)	118,554 (107,976–130,484)	−46	5597 (3914–7706)	5849 (3977–8048)	4	223,179 (216,642–229,864)	124,403 (113,390–136,107)	−44
Palestine	0.57	75,846 (68,219–83,975)	31,431 (24,502–39,774)	−59	3586 (2544–4831)	3266 (2255–4538)	−9	79,433 (71,341–87,766)	34,697 (27,702–43,034)	−56
Qatar	0.8	38,386 (28,814–50,253)	16,144 (11,147–22,773)	−58	3989 (2782–5439)	3441 (2440–4733)	−14	42,376 (32,600–54,473)	19,585 (14,459–26,356)	−54
Saudi Arabia	0.76	85,559 (77,918–93,903)	20,090 (17,725–22,742)	−77	3258 (2324–4357)	2864 (2028–3813)	−12	88,817 (81,094–97,189)	22,953 (20,425–25,781)	−74
Somalia	0.15	380,035 (359,276–402,133)	217,737 (188,533–253,963)	−43	6804 (4570–9591)	6786 (4565–9724)	0	386,839 (366,551–408,916)	224,523 (195,558–260,559)	−42
Sudan	0.43	270,463 (252,550–290,131)	102,900 (79,559–135,299)	−62	7221 (4932–9990)	6213 (4235–8569)	−14	277,684 (259,409–297,220)	109,112 (85,741–141,261)	−61
Syria	0.58	76,228 (69,751–83,006)	37,424 (27,052–47,636)	−51	4229 (2959–5715)	8398 (4695–14,357)	99	80,457 (73,755–87,715)	45,823 (35,144–57,195)	−43
Tunisia	0.65	86,176 (81,431–91,325)	24,401 (20,379–29,206)	−72	4185 (2932–5763)	3352 (2358–4636)	−20	90,362 (85,673–95,668)	27,753 (23,536–32,514)	−69
United Arab Emirates	0.88	44,587 (25,858–70,617)	9635 (5545–15,958)	−78	4707 (3207–6428)	3885 (2685–5325)	−17	49,294 (30,621–75,379)	13,520 (9078–19,664)	−73
Yemen	0.41	262,432 (252,686–272,966)	97,450 (86,201–109,915)	−63	5938 (4095–8071)	9469 (5970–15,234)	59	268,369 (258,479–278,816)	106,919 (94,845–120,445)	−60

95% uncertainty intervals are provided in parentheses

*YLDs* years lived with disability, *YLLs* years of life lost, *DALYS* disability-adjusted life-years. (Global Burden of Disease 2015 Study, Global, Eastern Mediterranean Countries, 1990–2015)

### YLDs

YLDs in the EMR did not track the global trend from 1990 to 2015. The under-5 YLD rate decreased by 0.6% in the EMR compared to 10.0% globally (Table 3). Five countries in the EMR had increased YLD rates (Table 3). Syria had the largest increase, 99%, followed by Yemen with a 59% increase. This increase was driven primarily by war in both countries, where it accounted for 52.4% of total YLDs in Syria and 36.9% in Yemen. The largest decrease in YLD rate was in Lebanon, a 43% decrease from 6804 (4457–10,960) to 3878 (2676–5307) per 100,000 population under 5.

### DALYs

In 2015, there were 69,297,241 under-5 DALYs in the EMR, which constituted 30.2% of total DALYs in the region for all ages. From 1990 to 2015, the under-5 DALY rate in the EMR decreased by 52.8%, the same as the decrease in the global rate (Table 3). For all countries, this decrease in the DALY rate was driven primarily by a decrease in the YLL rate (Table 3). Iran had the largest decrease in DALY rate, 79%, from 137,881 (122,316–155,406) in 1990 to 29,140 (22,262–37,880) per 100,000 population under 5 in 2015. The smallest decreases were in Kuwait (40%) and Somalia (42%).

## Discussion

Our study shows that progress across the region for child survival remains uneven, and total deaths for children under 5 decreased in the EMR at a slower rate than globally. Our study showed large variation in the burden by countries of the region, with about 80% of under-5 deaths occurring in six countries of the region (Afghanistan, Pakistan, Somalia, South Sudan, Sudan, and Yemen), and three countries (Sudan, Afghanistan, and Pakistan) among the 10 countries with the highest child mortality in the world (GBD 2015 Mortality and Causes of Death Collaborators 2016).

Although the top five causes of under-5 mortality—namely neonatal preterm birth complications, neonatal encephalopathy, lower respiratory tract infections (LRI), congenital defects, and diarrheal diseases—were the same globally and in the EMR, the early neonatal mortality burden still poses a huge problem in the region. The decrease in the EMR countries has been the smallest compared to other regions in the world between 1990 and 2015.

War and legal intervention ranked as the ninth cause of death in children under 5 years of age in the EMR, compared to 25th globally in 2015. This finding highlights the consequences of recent conflicts and political unrest in the region, and the wars that followed (Institute for Health Metrics and Evaluation 2017). The EMR also now carries the largest burden of displaced populations globally. Out of a total of 50 million refugees and internally displaced persons (IDPs) worldwide, more than 29 million (9 million refugees and 20 million IDPs) came from the region (Mokdad et al. 2016). The impact of these emergencies on public health is profound and affects both the displaced populations and host communities and usually results in food insecurity, lack of access to sanitation and health care facilities, and inadequate care. Conflicts also disrupt family, which further exacerbates child morbidity and mortality burden due to unhealthy environments, spread of disease, and decreased quantity and quality of food intake (WHO EMRO 2015).

Conflict also deteriorates child health by increasing the incidence of sexual violence against women and children. Higher rates of rape, sexually transmitted diseases, unwanted pregnancies, and unsafe abortions have been documented in previous conflicts (Akseer et al. 2015).

Poverty and economic inequity are also important determinants of child health in the EMR. A meta-analysis examined the association of poverty with infant mortality in the EMR countries and suggested that there is a significantly increased mortality risk in infants born in poor households. The results suggest that policies aimed at poverty alleviation and female literacy will substantially contribute to a decrease in infant mortality in the EMR (Cottingham et al. 2008).

Child marriage is highly prevalent in the EMR. A report showed that approximately 25% of all girls were married before the age of 18 years in 15 countries in the region (The World Bank). In four countries, Afghanistan, Somalia, Sudan, and Yemen, the rate is estimated to be as high as 50% (The World Bank). In addition, illiteracy, especially among young females, is a common problem in the EMR. The literacy rate among females older than 15 years is approximately 80% in the EMR on average, but it is estimated to be around 67% for Morocco, 66% for Yemen, 61% for Sudan, 55% for Pakistan, and 32% for Afghanistan (The World Bank).

Our findings showed that while YLLs and DALYs followed the global trend of decrease from 1990 to 2015, YLDs in the EMR did not decrease during this period, which demonstrates the lack of improvement in socioeconomic conditions, in addition to the lack of improvement in treatments and health care facilities.

Worldwide, successes in decreasing child mortality have been attributed to rising levels of income per person (Jahan 2008; O’Hare et al. 2013); higher education, especially in women of reproductive age (Preston 1975; Gakidou et al. 2010); lower fertility rates; and strengthened public health programs.

In the EMR, action must be taken immediately to save children’s lives by expanding effective preventive and curative interventions. The health interventions needed to address the major causes of neonatal death generally differ from those needed to address other under-5 deaths, and are closely linked to maternal health. Antenatal care, delivery in a health facility attended by a skilled birth attendant, and newborn care are all essential public health measures that need to be strengthened in the EMR. In addition, global policy changes, like prevention of war and peaceful resolutions of conflicts to improve the well-being of children.

More than half of under-5 child deaths are due to diseases that are preventable and treatable through good nutrition and simple, affordable interventions. For some of the most deadly childhood diseases, such as measles, polio, diphtheria, tetanus, pertussis, pneumonia due to *Haemophilus influenza* type B and *Streptococcus pneumoniae,* and diarrhea due to rotavirus, vaccines are available and can protect children from illness and death (Fuchs et al. 2010). Strengthening health systems with a focus on delivery strategies and mechanisms for scaling up coverage to provide such interventions to all children is crucial to accelerate progress in improving child health in the EMR.

Health education programs, including providing information and confronting cultural and religious barriers toward utilization of family planning services, are crucial to decrease child mortality rates in the EMR. Birth spacing, decreasing the rate of high-risk pregnancies, and delaying the age of marriage, in addition to literacy, have been found to be associated with child health and survival (UNICEF 2005; Grown et al. 2005; Jain and Kurz 2007; Bhutta et al. 2013, 2014; Nasrullah et al. 2014). In addition, special care and protection should be given to vulnerable populations in war times, as well as secure shelter, food, and access to health care to prevent the devastating effects of these emergencies on child health.

Study Limitations: While our paper reports important information using the GBD methodology, this information has wide uncertainty due to absence of data or data with poor quality, and possible bias from modeling. Despite such shortcoming in the estimates produced, it provides estimates to EMR countries that could be a baseline to gauge progress of interventions. The methodology used makes the estimates comparable across countries. The EMR is going through chronic and acute turmoil that makes it difficult to observe any improvement in the future.

## Conclusion

In spite of the global achievements in improving child survival across geographies, the pace of progress was slow and uneven in the EMR. Our findings reinforce the imperative need for intensive and accelerated action to decrease the burden of child morbidity and mortality in the EMR. Ministries of health, non-governmental organizations, and civic society in the region need to rise to the challenge and accelerate the pace of progress toward decreasing the unacceptably high mortality numbers among children under 5 years of age in the region. Political awareness, commitment, and leadership are needed to ensure that child health receives the attention and resources needed to end preventable child deaths.

## Electronic supplementary material

Below is the link to the electronic supplementary material.

Supplementary material 1 (DOCX 34 kb)

Supplementary material 2 (XLSX 26 kb)
