# Supplementary material for: Neonatal, infant, and under-5 mortality and morbidity burden in the Eastern Mediterranean region: findings from the Global Burden of Disease 2015 study
Source: Int J Public Health. 2017 Aug 3;63(Suppl 1):63–77. doi: 10.1007/s00038-017-0998-x (PMC5702263; doi:10.1007/s00038-017-0998-x)

International Journal of Public Health

Electronic Supplementary Material

**Article title:**

Neonatal, infant, and under-5 mortality and morbidity burden in the Eastern Mediterranean Region: Findings from the Global Burden of Disease 2015 study

**Authors:**

GBD 2015 Eastern Mediterranean Region Neonatal, Infant, and under-5 Mortality Collaborators

**Corresponding author:**

Ali H. Mokdad

Institute for Health Metrics and Evaluation, University of Washington, Seattle, WA, United States

Email: [mokdaa@uw.edu](mailto:mokdaa@uw.edu)

e-Table 1: Total deaths and mortality rates in the EMR and globally, subgroups of under-5, 1990–2015. Deaths are in thousands; rates are per 1,000 live births. 95% uncertainty intervals are provided in parentheses. (Global Burden of Disease 2015 study, Global, Eastern Mediterranean Region, 1990–2015)

|  | **Year** | **Early neonatal (0-6 days)** | | **Late neonatal (7-27 days)** | | **Post-neonatal (28 days–12 months)** | | **Child (12–59 months)** | | **Under-5 (0–59 months)** | |
| --- | --- | --- | --- | --- | --- | --- | --- | --- | --- | --- | --- |
|  |  | **Deaths** | **Rate** | **Deaths** | **Rate** | **Deaths** | **Rate** | **Deaths** | **Rate** | **Deaths** | **Rate** |
| **Eastern  Mediterranean  Region** | 1990 | 379.5 (361.6 – 396.7) | 27.7 (26.4 – 28.9) | 157.6 (153.0 – 162.5) | 11.5 (11.2 – 11.9) | 412.3 (390.2 – 434.0) | 30.1 (28.5 – 31.7) | 329.5 (297.5 – 362.8) | 24.0 (21.7 – 26.5) | 1278.8 (1251.9 – 1307.3) | 93.3 (91.3 – 95.4) |
|  | 1995 | 352.2 (335.9 – 367.9) | 25.7 (24.5 – 26.8) | 135.2 (130.5 – 139.5) | 9.8 (9.5 – 10.2) | 349.4 (331.0 – 368.8) | 25.5 (24.1 – 26.9) | 272.3 (242.6 – 300.7) | 19.8 (17.7 – 21.9) | 1109.1 (1084.9 – 1133.4) | 80.8 (79.1 – 82.6) |
|  | 2000 | 326.8 (311.8 – 341.7) | 23.6 (22.5 – 24.7) | 118.2 (113.7 – 122.6) | 8.5 (8.2 – 8.8) | 300.7 (284.2 – 317.6) | 21.7 (20.5 – 22.9) | 231.4 (205.7 – 257.8) | 16.7 (14.8 – 18.6) | 977.1 (953.8 – 999.7) | 70.5 (68.8 – 72.1) |
|  | 2005 | 315.4 (299.9 – 329.6) | 21.5 (20.4 – 22.4) | 107.8 (102.9 – 112.4) | 7.3 (7.0 – 7.6) | 267.0 (251.6 – 282.9) | 18.2 (17.1 – 19.2) | 202.3 (180.1 – 226.8) | 13.8 (12.3 – 15.4) | 892.4 (868.0 – 916.8) | 60.7 (59.1 – 62.4) |
|  | 2010 | 313.6 (297.6 – 329.7) | 19.3 (18.3 – 20.3) | 101.1 (95.5 – 106.4) | 6.2 (5.9 – 6.5) | 246.1 (228.3 – 265.8) | 15.1 (14.0 – 16.3) | 174.5 (152.8 – 196.0) | 10.7 (9.4 – 12.1) | 835.3 (801.5 – 869.3) | 51.4 (49.3 – 53.5) |
|  | 2015 | 294.6 (276.4 – 313.0) | 17.2 (16.2 – 18.3) | 90.7 (84.0 – 97.2) | 5.3 (4.9 – 5.7) | 211.8 (194.9 – 229.8) | 12.4 (11.4 – 13.4) | 158.7 (138.0 – 180.0) | 9.3 (8.1 – 10.5) | 755.8 (712.1 – 801.6) | 44.2 (41.6 – 46.9) |
| **Global** | 1990 | 3288.2 (3225.0 – 3349.1) | 23.6  (22.6 – 24.6) | 1264.7  (1250.5 – 1279.3) | 9.3  (9.0 – 9.7) | 3784.5  (3693.5 – 3877.4) | 28.1 (26.2 – 29.9) | 3782.7  (3637.2 – 3933.8) | 28.9 (26.6 – 31.7) | 12120.1  (12010.8 – 12239.5) | 87.1  (84.5 – 90.1) |
|  | 1995 | 2970.8  (2910.8 – 3030.0) | 22.4  (21.5 – 23.3) | 1079.3  (1067.2 – 1091.5) | 8.3  (8.0 – 8.6) | 3285.5  (3200.4 – 3372.3) | 25.4  (23.9 – 26.9) | 3291.7  (3160.1 – 3422.1) | 25.4 (23.4 – 27.5) | 10627.2  (10534.2 – 10728.5) | 79.2  (77.1 – 81.3) |
|  | 2000 | 2721.6 (2666.4 – 2773.4) | 20.9  (20.0 – 21.6) | 927.5  (915.8 – 939.0) | 7.3  (7.0 – 7.5) | 2843.1  (2763.3 – 2918.7) | 22.5  (21.2 – 23.8) | 2810.3  (2688.1 – 2935.6) | 22.4  (20.7 – 24.3) | 9302.5  (9218.1 – 9386.2) | 71.1  (69.3 – 73.0) |
|  | 2005 | 2492.3  (2439.1 – 2543.3) | 18.6  (17.8 – 19.4) | 795.7  (785.0 – 807.6) | 6.1  (5.8 – 6.3) | 2389.6  (2321.1 – 2460.2) | 18.4  (17.3 – 19.5) | 2319.2  (2211.2 – 2428.0) | 18.3  (16.9 – 19.9) | 7996.7  (7921.6 – 8081.5) | 60.0  (58.2 – 62.0) |
|  | 2010 | 2264.3  (2214.9 – 2314.3) | 16.4 (15.6 – 17.2) | 686.9  (674.8 – 700.2) | 5.1  (4.8 – 5.4) | 2022.5  (1958.0 – 2083.5) | 15.0  (13.9 – 16.1) | 1902.2  (1803.8 – 1996.8) | 14.5  (13.1 – 15.9) | 6875.8  (6777.7 – 6980.6) | 50.0  (47.3 – 52.7) |
|  | 2015 | 2034.2  (1983.0 – 2082.9) | 14.5 (13.4 – 15.6) | 587.2  (570.6 – 604.0) | 4.2  (3.8 – 4.7) | 1678.0  (1617.9 – 1741.5) | 12.2  (10.9 – 13.6) | 1521.4  (1425.6 – 1620.6) | 11.2  (9.8 – 12.8) | 5820.9  (5673.3 – 5965.1) | 41.4  (37.9 – 45.5) |

| **Country** | **SDI** | **Mortality rate (per 100,000 under-5 population)** | | |
| --- | --- | --- | --- | --- |
|  |  | **Observed** | **Expected on the basis of SDI alone** | **Observed/expected ratio** |
| Global | 0.64 | 866.8 (844.8 – 888.3) | 973.9 | 0.89 |
| Eastern Mediterranean Region | 0.55 | 941.8 (887.2 – 998.7) | 1147.2 | 0.82 |
| Afghanistan | 0.29 | 1774.3 (1501.1 – 2101.5) | 2540.9 | 0.70 |
| Bahrain | 0.78 | 132.5 (112.7 – 155.2) | 212.5 | 0.62 |
| Djibouti | 0.46 | 1379.9 (1165.9 – 1657.9) | 1262.8 | 1.09 |
| Egypt | 0.62 | 453.3 (341.4 – 596.5) | 655.9 | 0.69 |
| Iran | 0.72 | 293.7 (216.0 – 392.2) | 336.4 | 0.87 |
| Iraq | 0.58 | 584.8 (504.7 – 685.4) | 807.6 | 0.72 |
| Jordan | 0.7 | 305.0 (266.3 – 354.5) | 400.1 | 0.76 |
| Kuwait | 0.86 | 170.4 (134.8 – 213.9) | 105.5 | 1.62 |
| Lebanon | 0.75 | 180.8 (132.0 – 251.4) | 276.7 | 0.65 |
| Libya | 0.64 | 344.8 (263.9 – 446.3) | 530.5 | 0.65 |
| Morocco | 0.5 | 459.5 (368.4 – 571.8) | 1099.7 | 0.42 |
| Oman | 0.73 | 199.4 (172.6 – 228.9) | 310.6 | 0.64 |
| Pakistan | 0.47 | 1376.4 (1253.2 – 1515.2) | 1292.3 | 1.07 |
| Palestine | 0.57 | 365.0 (284.6 – 461.8) | 836.0 | 0.44 |
| Qatar | 0.8 | 187.6 (129.6 – 264.6) | 179.0 | 1.05 |
| Saudi Arabia | 0.76 | 233.6 (206.1 – 264.5) | 253.2 | 0.92 |
| Somalia | 0.15 | 2542.4 (2199.1 – 2965.1) | 4384.8 | 0.58 |
| Sudan | 0.43 | 1198.4 (926.1 – 1576.9) | 1464.3 | 0.82 |
| Syria | 0.58 | 437.8 (315.8 – 557.8) | 736.7 | 0.59 |
| Tunisia | 0.65 | 283.7 (236.9 – 339.6) | 526.6 | 0.54 |
| United Arab Emirates | 0.88 | 112.0 (64.5 – 185.6) | 96.9 | 1.16 |
| Yemen | 0.41 | 1135.4 (1003.6 – 1281.1) | 1543.3 | 0.74 |

e-Table 2: Socio-demographic Index (SDI), observed and expected under-5 mortality rates in 2015. 95% uncertainty intervals are provided in parentheses. (Global Burden of Disease 2015 study, Global, Eastern Mediterranean Region, 2015)

e-Figure 1: Observed and expected based on Socio-demographic Index (SDI) under-5 mortality rates in the Eastern Mediterranean Region for all causes, 1990–2015 (Global Burden of Disease 2015 study, Eastern Mediterranean Region, 1990–2015)


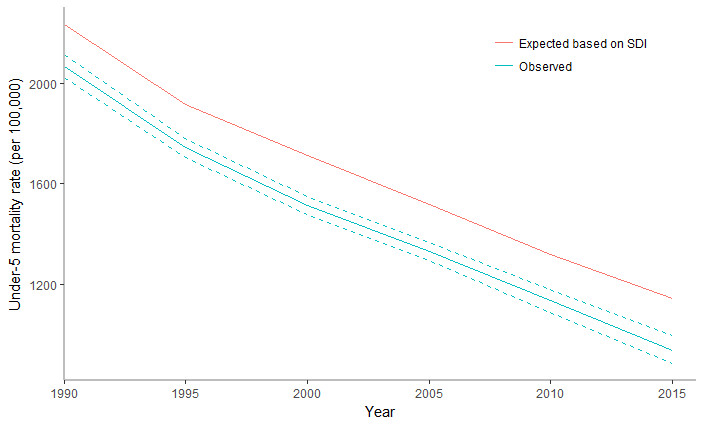

Supplement: Supplementary file 1 — Supplementary material 1 (DOCX 34 kb) [file 38_2017_998_MOESM1_ESM.docx]
